# Protocol for RETRO-TBI: A prospective cohort study of mild traumatic brain injury in older adults

**DOI:** 10.1371/journal.pone.0350552

**Published:** 2026-06-01

**Authors:** Jennifer S. Albrecht, Odessa Addison, Neeraj Badjatia, Mary K. Bahr-Robertson, Lynn M. Grattan, Ann L. Gruber-Baldini, Rosemary Kozar, Jay Magaziner, Denise L. Orwig, Michelle Newman, Deborah Stevens, Emerson M. Wickwire, Yuanyuan Liang

**Affiliations:** 1 Department of Epidemiology and Public Health, University of Maryland School of Medicine, Baltimore, Maryland, United States of America; 2 Department of Physical Therapy and Rehabilitation Science, University of Maryland School of Medicine, Baltimore, Maryland, United States of America; 3 Baltimore Veterans Affairs Geriatric Research, Education and Clinical Center, Baltimore, Maryland, United States of America; 4 Department of Neurology, University of Maryland School of Medicine, Baltimore, Maryland, United States of America; 5 Shock Trauma Center, University of Maryland School of Medicine, Baltimore, Maryland, United States of America; 6 Division of Pulmonary, Critical Care, and Sleep Medicine, Department of Medicine, University of Maryland School of Medicine, Baltimore, Maryland, United States of America; 7 Department of Psychiatry, University of Maryland School of Medicine, Baltimore, Maryland, United States of America; PLOS: Public Library of Science, UNITED KINGDOM OF GREAT BRITAIN AND NORTHERN IRELAND

## Abstract

Older adults experience the highest rates of traumatic brain injury (TBI) related hospitalizations and deaths of any age group, yet TBI remains understudied in this population. To improve understanding of recovery over the year following TBI among older adults, we designed the RETRO-TBI study. This manuscript reports the protocol for RETRO-TBI, a prospective cohort study of older adults (65 years and older) with mild TBI (mTBI) with planned enrollment of 250 participants. The study is designed to evaluate recovery across four key domains: physical function, cognitive function, psychological function, and sleep quality. Participants are followed for 12 months after injury, with in-home study visits conducted at approximately 2 weeks and 3, 6, and 12 months post-injury. Blood samples are collected at all visits. The specific aims are to: (1) identify trajectories of recovery in physical function and predictors of poorer physical recovery; (2) identify trajectories of recovery in cognitive function, psychological function, and sleep quality and predictors of poorer recovery in these domains; and (3) examine associations among recovery trajectories across domains. The RETRO-TBI study represents an important step in addressing the knowledge gap on recovery following TBI among older adults and is expected to result in identification of sub-groups of individuals more likely to have poor recovery, informing individualized treatment plans and development of future domain-based rehabilitation strategies. The study has several strengths including its focus on older adults, evaluation of recovery across four domains of function, and longitudinal assessments will permit evaluation of heterogeneity in recovery trajectories.

## 1. Introduction

Traumatic brain injury (TBI) is a common injury among older adults that results in over 123,000 hospitalizations and 485,000 emergency department visits annually in the United States [1,2]. Rates of TBI have increased rapidly over the last two decades among older adults [1–3], with falls the primary injury mechanism [4,5]. The majority of TBI among older adults are mild [4,5]. TBI, including mTBI, is associated with increased risk for neurodegenerative, neuropsychiatric, and sleep disorders and is a known risk factor for Alzheimer’s disease and related dementias (ADRD) [6–11]. TBI is also associated with disability, institutionalization, and increased mortality [12–15].

Although older adults experience the highest rates of TBI-related hospitalizations and deaths of any age group [1], TBI remains understudied in this population [16,17]. TBI mechanism, presentation, and recovery among older adults differ in multiple ways from younger adults, making generalizations from research in younger adults challenging [1,4,18–22]. For example, most older adults with TBI have a high comorbidity burden where a majority have several chronic conditions like ADRD, depression, diabetes, and cardiovascular disease [22–26]. This comorbidity burden and resulting polypharmacy complicate recovery following injury, which is slower than that observed in younger adults [4,19,20]. Still, the majority of older adults hospitalized with TBI spend most of their time at home and not in a hospital or care facility by six months post-injury [27].

To improve understanding of recovery following TBI among older adults, we designed a prospective cohort study. Results from this study are expected to advance understanding of recovery after TBI in older adults across physical, cognitive, psychological and sleep domains, facilitate early identification of patients at risk for poorer recovery trajectories, and inform development and timing of interventions to optimize outcomes. This manuscript presents the detailed protocol for the study, entitled RETRO-TBI.

## 2. Methods

### 2.1. Study design

RETRO-TBI is a prospective cohort study of older adults (65 years and older) with mild TBI (mTBI). The study is designed to evaluate recovery over the first year after injury across four key domains: physical function, cognitive function, psychological function, and sleep quality. We focused on mTBI because it is by far the most common type of TBI, comprising >90% of cases [4,28], yet it is the least studied in terms of recovery. Participants are followed for 12 months after injury, with study visits conducted at approximately 2 weeks and 3, 6, and 12 months post-injury (Table 1). The specific aims are to: (1) identify trajectories of recovery in physical function and predictors of poorer physical recovery; (2) identify trajectories of recovery in cognitive function, psychological function, and sleep quality and predictors of poorer recovery in these domains; and (3) examine associations among recovery trajectories across domains.

**Table 1:** RETRO-TBI assessments.

Functional Domain	2 Weeks	3 Months	6 Months	12 Months
***Cognitive***				
Short Informant Questionnaire on Cognitive Decline in the Elderly [40] (proxy)	X			
Repeatable Battery for the Assessment of Neuropsychological Status [30,31]	X	X	X	X
Frontal Assessment Battery (FAB) [41]	X	X	X	X
***Physical***				
Activities of Daily Living [42]	X	X	X	X
Instrumental Activities of Daily Living [43]	X	X	X	X
Short Performance Physical Battery [32]	X	X	X	X
Four-Step Square Test [33]	X	X	X	X
Glasgow Outcomes Scale-Extended [44]	X	X	X	X
***Psychological***				
Geriatric Depression Scale-15 [45]	X	X	X	X
Hospital Anxiety and Depression Scale- anxiety subscale [46]	X	X	X	X
Rivermead post-concussive symptoms questionnaire	X	X	X	X
3-Item Alcohol Use Disorders Identification Test Consumption [39]	X			
Ohio State University TBI-ID [37,47]	X			
***Sleep***				
Consensus Sleep Diary (1 week)	X	X	X	X
Actigraphy (1 week)	X			
Pittsburgh Sleep Quality Index [35]	X	X	X	X
Insomnia Severity Index [34]	X	X	X	X

### 2.2. Study setting and study population

Participants are recruited from the R Adams Cowley Shock Trauma Center and affiliated clinical settings within the University of Maryland system. The study focuses on older adults evaluated clinically for isolated mild TBI. Follow-up assessments are conducted in participants’ homes or current place of residence to reduce participant burden and minimize loss to follow-up.

Patients aged 65 years or older who are evaluated at the STC with isolated mild TBI within 72 hours of injury are eligible. Mild TBI is defined in accordance with the American Congress of Rehabilitation Medicine (ACRM) criteria [29] as a traumatically induced physiological disruption of brain function as manifested by at least one of the following: loss of consciousness (LOC) ≤30 minutes, post-traumatic amnesia (PTA) <24 hours, observed or self-reported transient confusion, disorientation, or impaired consciousness, or neurologic deficit as defined by a Glasgow Coma Scale (GCS) score of 13–14 on admission. GCS of 15 with no other evidence of mTBI is also accepted if a computed tomography image demonstrates an acute injury. Exclusion criteria include severe injury to other body regions, anticipation that the patient will not be ambulatory at 2-weeks post-injury, history of dementia as determined by patient/proxy report or medical history, non-ambulatory pre-injury, non-English-speaking, being a prisoner, or live >55 miles from the STC (regardless of recruitment location).

### 2.3. Recruitment and enrollment

Recruitment for RETRO-TBI began in May 2023. Eligible patients are contacted by RETRO-TBI research staff by phone or in person (depending on discharge status) within 48 hours of admission to confirm eligibility and ask about participation in the study. If an eligible participant is interested, the baseline assessment (two-weeks post-TBI) is scheduled. To ensure enrollment goals are achieved, we expanded recruitment to the University of Maryland Medical Center’s (UMMC) emergency department (ED) in July 2023 and expanded to six additional hospitals affiliated with the University of Maryland Medical System in mid-2025. In the UMMC ED, two research assistants screen patients aged 65 and older with history of a fall or head impact for possible TBI five days a week during working hours and provide a study pamphlet to possible participants. Study staff follow-up with a phone call to confirm inclusion criteria by asking a series of questions that mirror the ACRM criteria. Questions assess for presence of any LOC or PTA or symptoms, including confusion, headache, nausea, dizziness, light sensitivity, or feeling groggy. Exclusion criteria are identical. Recruitment from other UMMS hospitals relies on centralized daily screening of EPIC admission diagnoses for international classification of disease, version 10 (ICD-10) codes for TBI. To screen for possible TBI, an automated algorithm searches for ICD-10 codes S01, S02, S06, S07 and S09 among hospitalized patients aged 65 and older. Research staff then access the patient’s medical record to assess inclusion/exclusion criteria. Patients meeting this initial screen are mailed a study pamphlet. Six days later, research staff call the patient and verify they meet study inclusion/exclusion criteria before scheduling the baseline assessment. The University of Maryland School of Medicine Institutional Review Board granted approval for this minimal risk study and all associated recruitment activities (HP-00100973). Enrollment will be completed by October 2026 with completion of follow-up by October 2027.

### 2.4. Staff training

Two research staff were trained on phlebotomy and all assessments by experts in the field over the course of three months leading up to the start of recruitment. Administration and scoring of the Repeatable Battery for the Assessment of Neuropsychological Status (RBANS) [30,31] required multiple training sessions. Inter-rater concordance with the supervising neuropsychologist was established before independent administration. The research staff were trained on the Short Physical Performance Battery (SPPB) [32] and the Four-Step Square Test (FSST) [33] by a physical therapist. Training included ascertainment of whether the participant was safe to perform the assessment and use of the gait belt. Training on the sleep assessments, including the Insomnia Severity Index (ISI) [34], the Pittsburgh Sleep Quality Index (PSQI) [35], and instructions for wearing the Philips Actiwatch series actigraph and completing the sleep diary was provided by a board-certified sleep psychologist expert in sleep assessment and actigraphy. Training for all other assessments was provided by the PI. Before the start of enrollment, the research staff members practiced the entire process from evaluation to sign informed consent through the blood draw and all assessments four times. The two trained research staff assessors complete an entire assessment together twice annually to verify that study procedures are performed according to study protocol.

### 2.5. Study assessments and timing

RETRO-TBI has four study visits: 2 weeks and 3, 6, and 12 months following the date of mTBI. The baseline assessment can be completed between 11−21 days post-TBI at the participant’s home or current place of residence. Assessment at two weeks, rather than immediately post-injury, permits participants to stabilize from the acute hospitalization episode and is consistent with the timeline used in the TRACK-TBI study [36]. Later assessments have a + /- 2 week window for completion. The informed consent process occurs at the baseline assessment before any study procedures take place. All participants are evaluated for ability to provide written informed consent using a 5-item screening instrument that assesses understanding of study risks, expectations, how to exit the study and what to do if they experience discomfort during study visits or procedures. Correct answers are required on all questions before proceeding to written informed consent and study procedures. Proxy respondents are not used.

Study assessments and timing are listed in Table 1 and described below. The baseline assessment also includes demographic and contact information, 12-month fall and lifetime TBI history (Ohio State University TBI-ID), description of the events leading up to the injury, education, current employment status, and self-reported comorbidities consistent with National Institute for Neurological Disorders and Stroke TBI common data elements [36–38]. We later added questions on substance use, including the three item Alcohol Use Disorders Identification Test Consumption (AUDIT) [39], questions about use of illicit or non-prescription drugs, painkillers, and cannabis, and two questions on food security. A blood draw is performed at the baseline assessment but was later added to the remaining three assessments. Participants are offered breaks during the assessment as needed and the visit can be broken into two sessions with a maximum of two days between visits if requested. Compensation of $50 (gift card) for each assessment is offered ($200 total per patient for the four assessments). Data from study assessments is directly uploaded to a REDCap database via iPad.

### 2.6. Measures

#### 2.6.1. Cognitive function.

The presence of unreported or unrecognized cognitive impairment pre-injury is assessed using the Informant Questionnaire on Cognitive Decline in the Elderly (IQCODE). The IQCODE is a 16-item measure administered to proxy respondents to assess cognitive decline over the past 10-years [40]. Cognitive function is assessed using the RBANS and the Frontal Assessment Battery (FAB). The RBANS is a brief, individually administered series of cognitive measures that allows tracking of cognitive status over time and has four equivalent forms that assess immediate and delayed memory, attention, language, constructional praxis, and visuospatial skills [30,31]. It has been demonstrated to be sensitive in patients with TBI and in a geriatric population [48,49]. The RBANS is administered using licensed software that includes image capture and recording functions via iPad. The FAB is a brief neuropsychological battery designed to assess executive functions at bedside [41]. It comprises six cognitive tasks that measure conceptualization, mental flexibility, motor programing, sensitivity to interference, inhibitory control, and environmental autonomy.

#### 2.6.2. Physical function.

Two objective and two subjective assessments of physical function are assessed. The SPPB is used to assess measures of lower extremity function including balance, chair stands and a timed 4-meter walk, and is associated with disability and mortality in older adults [50,51]. Lower extremity function is highly predictive of falls, the primary cause of TBI among older adults [1,52,53]. The FSST is used to assess dynamic balance and obstacle negotiation, both important components of fall risk [33,54,55]. The FSST is a timed test of multidirectional stepping motions that assesses the ability of an individual to dynamically maintain an upright posture while changing directions and weight shifting, and has excellent predictive ability to identify individuals at risk of falls [33]. We also assess disability via self-report using Activities of Daily Living (ADLs) and the Instrumental Activities of Daily Living (IADLs) surveys [42,43]. Finally, we assess self-reported overall recovery using the Glasgow Outcomes Scale-Extended (GOSE) [44].

#### 2.6.3. Sleep.

Consistent with consensus recommendations for measurement of sleep in mTBI [56], we employ objective and subjective measures of sleep. Subjective measures include modified consensus sleep diaries [57], the PSQI and the ISI [34,35]. Primary outcomes of the modified consensus sleep diary will include standard sleep continuity parameters (habitual total sleep time, sleep onset latency, wake after sleep onset, sleep efficiency, and sleep quality). The PSQI is a 19-item questionnaire that measures sleep quality while the ISI is a 7-item scale that measures perceived insomnia severity [34,35]. Both measures have been validated in TBI populations and the PSQI has been used to track sleep disturbances over time in mTBI patients [58–60]. In addition to these subjective measures of sleep, we also include actigraphy, a validated proxy for sleep that has been used in numerous studies of mTBI [61–63]. Participants wear a Philips Actiwatch Spectrum Plus on their non-dominant hand for 1 week, with concurrent sleep diary, during the baseline assessment period. Complementing the sleep diaries, actigraphy at 2-weeks post-injury will provide objective measures of habitual total sleep time, sleep onset latency, wake after sleep onset, as well as secondary variables.

#### 2.6.4. Psychological function.

The Geriatric Depression Scale, a 15-item self-report measure that has been validated in hospitalized older adults, is used to assess symptoms of depression [45]. To measure symptoms of anxiety, we use the anxiety sub-scale of the Hospital Anxiety and Depression Scale (HADS-A) [46,64]. The HADS-A is a seven-item measure scored from 0–21 that has been validated in TBI patients and has good sensitivity to detect anxiety compared to a structured clinical interview [65,66]. The Rivermead post-concussion symptoms questionnaire evaluates symptoms of concussion [67].

#### 2.6.5. Medical chart review.

Clinical and injury-related information, including hospital course, GCS, CT scan results, pre-existing comorbid conditions including history of dementia and other neurological conditions, home medications and discharge disposition, is obtained through medical chart abstraction using a standard abstraction form.

The Rotterdam CT score is calculated from the initial non-contrast head CT following the methodology described by Maas et al. (2005) [68], which comprises four independently graded components: basal cistern status (0 = normal, 1 = compressed, 2 = absent), midline shift (0 = no shift or ≤5 mm, 1 = > 5 mm), epidural mass lesion (0 = present, 1 = absent), and intraventricular or subarachnoid hemorrhage (0 = absent, 1 = present). These component scores are summed and 1 is added to the total, yielding an ordinal score ranging from 1 (normal-appearing scan) to 6 (worst findings). The presence of subdural hematoma is evaluated separately on the same initial CT as a dichotomous variable (present vs. absent).

### 2.7. Statistical analysis

#### 2.7.1. Overview.

All statistical analyses will be performed using Stata/SE version 19. Statistical tests will be two-sided with a significance level of 0.05. Prior to analysis, data will be reviewed for completeness, range errors, outliers, and internal inconsistencies. Continuous variables will be summarized using means and standard deviations or medians and interquartile ranges, as appropriate, and categorical variables using counts and percentages. The distribution of continuous variables will be assessed graphically and analytically, and transformations will be considered when needed to satisfy model assumptions. Baseline characteristics will be summarized for the overall cohort and, where relevant, by follow-up status and trajectory group membership.

Group-based trajectory modeling (GBTM) will be used to identify distinct longitudinal patterns of recovery over 12 months after mTBI. Four assessments are planned at approximately 2 weeks and 3, 6, and 12 months after injury. Separate trajectory models will be fit for each primary domain-specific outcome: SPPB for physical function, RBANS total score for cognition, GDS score for psychological function, and sleep duration derived from the sleep diary for sleep. Additional outcomes within each domain will be evaluated in secondary analyses. For each GBTM, candidate models with varying numbers of trajectory groups and polynomial forms will be compared. Final model selection will be guided by a combination of Bayesian Information Criterion, clinical interpretability, adequacy of group size, posterior probabilities of assignment, and consistency with recommended diagnostic criteria for trajectory modeling. Participants will be assigned to the trajectory group for which they have the highest posterior probability. Identified trajectories will be displayed graphically and summarized descriptively.

After trajectory groups are identified, we will examine baseline demographic, clinical, and injury-related predictors of group membership. Variables of interest include age, sex, pre-injury cognitive status, injury severity measures, CT abnormalities, comorbidity burden, and selected baseline functional or sleep measures. Associations with trajectory group membership will first be examined descriptively and in bivariate analyses. Multivariable multinomial logistic regression models will then be used to estimate associations between candidate predictors and trajectory group membership. Because this is an observational study with a moderate expected sample size after attrition, multivariable models will emphasize parsimonious adjustment and clinically important predictors.

Missing longitudinal outcome data will be described at each time point. GBTM accommodates incomplete repeated-measures outcome data under standard model assumptions, allowing participants with partial follow-up to contribute information. Patterns of missingness and loss to follow-up will be examined, and sensitivity analyses will be considered if dropout appears to differ systematically by baseline characteristics. Missing covariate data, if present, will be handled using appropriate methods such as indicator categories or multiple imputation, depending on the extent and nature of missingness.

#### 2.7.2. Sample size considerations.

RETRO-TBI sample size was estimated using pilot data from the R Adams Cowley Shock Trauma Center (STC) at the University of Maryland, Baltimore, and projected a conservative 45% enrollment rate. On this basis, we projected enrollment of approximately 8 participants per month over a 34-month study period, including an 8-month start-up phase to hire and train staff and a 6-month close-out period. Although this projection suggested a maximum sample of 272 (8 × 34) participants, we conservatively set the enrollment target at 250. Assuming that 20% of participants will not complete at least two of the four longitudinal assessments, 200 participants are expected to contribute to the GBTM analyses. Given that GBTM can accommodate missing data and is generally supported with sample sizes of at least 100 [69], the planned sample size is adequate for the proposed GBTM analyses and permits preliminary modeling of predictors of poor recovery.

For bivariate analyses examining associations between categorical predictors and trajectory group membership, power was estimated using chi-square tests. For example, comparison of two age groups (65–79 vs. ≥ 80 years) across three SPPB trajectory groups would involve 2 degrees of freedom. With a sample size of 200, a two-sided alpha of 0.05, and contingency tables with 2–10 degrees of freedom, the study will have 80% power to detect effect sizes (Cohen’s *w*) of approximately 0.22 to 0.29, representing small-to-moderate associations. According to Cohen’s conventions, values of *w* of 0.10, 0.30, and 0.50 correspond to small, medium, and large effect sizes, respectively.

#### 2.7.3. Aim 1.

Aim 1: Assess recovery of physical functioning and identify predictors of poor recovery.

The primary outcome for Aim 1 is the SPPB score, assessed at approximately 2 weeks and 3, 6, and 12 months after injury. Recovery trajectories in physical functioning will be modeled using GBTM to identify distinct subgroups of participants with similar longitudinal patterns of SPPB performance over follow-up. Secondary physical outcomes, including ADLs, IADLs, and FSST performance, will be examined using the same trajectory-modeling framework as secondary analyses.

After SPPB trajectory groups are identified, baseline demographic and clinical characteristics will be compared across groups using appropriate descriptive and inferential methods. Continuous variables will be compared using analysis of variance or Kruskal-Wallis tests, as appropriate, and categorical variables using chi-square or Fisher’s exact tests. Multinomial logistic regression will then be used to identify predictors of physical function trajectory group membership. Predictors of primary interest include age, sex, injury severity measures, head Abbreviated Injury Scale score, acute CT findings, pre-injury functional status, comorbidity burden, and other clinically relevant baseline characteristics. Exploratory stratified analyses by age group (65–79 vs. ≥ 80 years) and sex will be performed if cell sizes permit stable estimation.

#### 2.7.4. Aim 2.

Aim 2: Assess recovery of cognitive and psychological functioning and sleep quality and identify predictors of poor recovery.

For cognition, the primary outcome is the RBANS total score; for psychological function, the primary outcome is the GDS score; and for sleep, the primary outcome is sleep duration derived from the sleep diary. Separate GBTMs will be fit for each of these three outcomes to identify distinct trajectories of cognitive recovery, psychological recovery, and sleep recovery over 12 months.

Secondary analyses within Aim 2 will evaluate other measures collected in these domains, including the Frontal Assessment Battery, anxiety symptoms measured by the Hospital Anxiety and Depression Scale anxiety subscale, PTSD symptoms measured by the PTSD Checklist for DSM-5, sleep quality measured by the Pittsburgh Sleep Quality Index, insomnia severity measured by the Insomnia Severity Index, and other diary- or actigraphy-derived sleep parameters as appropriate.

For each primary Aim 2 outcome, baseline characteristics will be summarized by trajectory group membership and compared across groups. Multinomial logistic regression models will be used to identify predictors of poorer trajectory group membership for each domain. Predictors of interest will include age, sex, education, pre-injury cognition assessed by IQCODE, injury severity measures, CT abnormalities, baseline functional status, and selected sleep-related or psychological variables, depending on the domain being modeled. To preserve model stability, multivariable analyses will emphasize a limited number of clinically relevant covariates.

#### 2.7.5. Aim 3.

Aim 3 (exploratory): Evaluate associations between recovery trajectories across functional domains.

To assess whether recovery in one domain is associated with recovery in another, we will compare trajectory group membership across the four primary domain-specific outcomes. For example, physical function trajectory group membership will be compared with trajectory group membership in cognitive, psychological, and sleep domains. Associations between pairs of trajectory group memberships will first be assessed using chi-square tests. We will then fit multinomial logistic regression models in which trajectory group membership in one domain is modeled as a function of trajectory group membership in another domain, adjusting for age and sex.

These analyses are exploratory and are intended to generate insight into whether recovery patterns cluster across domains in ways that may inform future multidomain intervention studies. Because the number and size of trajectory groups may differ by outcome, these analyses will be interpreted as hypothesis-generating rather than confirmatory.

## 3. Discussion

Although older adults experience the highest rates of TBI-related hospitalization and mortality, they remain underrepresented in research [4,16,17]. Understanding of how older adults recover from mTBI is needed to guide targeted rehabilitation, care planning, and promote optimal recovery among older adults with TBI, yet it is severely lacking in the literature.

The RETRO-TBI study represents an important step in addressing this knowledge gap. Results will provide detailed information on longitudinal recovery in cognitive, physical, and psychological functioning and sleep quality over the year following mTBI among older adults for the first time. By documenting recovery across four critical domains of functioning, results from this study are expected to have broad impact on research in older adult TBI, permitting in depth focus on specific domains of function. Examples of future studies include development of interventional studies in cognitive and physical rehabilitation, sleep disorders, and psychological treatment. Biospecimens will be available for future analysis of TBI and ADRD biomarkers and their associations with recovery.

RETRO-TBI is expected to result in identification of sub-groups of individuals more likely to have poor recovery. This information will be particularly useful for targeting individualized treatment plans and informing development of future domain-based rehabilitation strategies. By identifying interactions between recovery domains, we hope to gain understanding on how recovery in a single domain impacts recovery in other domains. Such information will provide insight into mechanisms of recovery.

The study has several strengths. First, it focuses specifically on older adults, a group underrepresented in prior TBI research. Second, it evaluates recovery across physical, cognitive, psychological, and sleep domains, allowing a more comprehensive characterization of post-injury function than studies relying solely on global outcome scales. Third, repeated assessments over 12 months following mTBI permit evaluation of heterogeneity in recovery trajectories. Fourth, a diverse group of mTBI patients are included from multiple hospitals. Fifth, in-home visits reduce loss-to follow-up (relative to requiring a trip to the study site) while two interviewers over the course of the study reduces interviewer-introduced bias. Quality control procedures help ensure valid data collection. Lastly, the proposed group-based trajectory models will identify distinct recovery trajectories, permitting targeting of individuals vulnerable to poor recovery.

Limitations include exclusion of individuals who are not treated at recruitment locations. This exclusion has two possible effects. First, our recruitment locations are located primarily in urban areas in one state, possibly limiting generalizability. However, individuals in the study can live up to 55 miles from STC, encompassing rural areas of Maryland. Second, individuals with very mTBI not requiring medical evaluation are likely to be excluded. This interesting group should be the focus of a future study. RETRO-TBI excludes individuals with pre-existing ADRD. Given prior studies suggesting that one-third of older Medicare beneficiaries hospitalized with TBI have an ADRD diagnosis, this group of individuals also requires focused future research effort [70]. Due to delayed implementation of blood draws at all study visits, we will have fewer samples at later timepoints. Because the study is observational, identified associations between baseline characteristics and recovery trajectories should not be interpreted causally. As in all longitudinal cohort studies, loss to follow-up and missing data may affect precision and introduce bias. Despite these limitations, RETRO-TBI is expected to provide important new information on multidomain recovery after mTBI in older adults. By identifying distinct trajectories of recovery and the characteristics associated with poorer outcomes, the study will provide a foundation for risk stratification, targeted rehabilitation, and future interventions designed to optimize recovery in this understudied population.

In conclusion, RETRO-TBI is a prospective cohort study of 250 older adults with mTBI designed to characterize multidomain recovery over the year following injury. The study is expected to expand knowledge of how older adults recover from a common fall-related injury and guide multiple future studies to optimize recovery following TBI.

## References

[1] Taylor CA, Bell JM, Breiding MJ, Xu L. Traumatic brain injury-related emergency department visits, hospitalizations, and deaths - United States, 2007 and 2013. Morb Mortal Wkly Rep Surveill Summ. 2017;66(9):1–16. doi: 10.15585/mmwr.ss6609a1 28301451 PMC5829835

[2] Albrecht JS, Hirshon JM, McCunn M, Bechtold KT, Rao V, Simoni-Wastila L, et al. Increased rates of mild traumatic brain injury among older adults in US emergency departments, 2009-2010. J Head Trauma Rehabil. 2016;31(5):E1-7. doi: 10.1097/HTR.0000000000000190 26479396 PMC4834064

[3] Peterson AB, Daughterty J, Breiding MJ. Surveillance report of traumatic brain injury-related emergency department visits, hospitalizations, and deaths—United States, 2014. In: Services USDoHaH, editor. United States: Centers for Disease Control and Prevention; 2019.

[4] Gardner RC, Dams-O’Connor K, Morrissey MR, Manley GT. Geriatric traumatic brain injury: epidemiology, outcomes, knowledge gaps, and future directions. J Neurotrauma. 2018;35(7):889–906. doi: 10.1089/neu.2017.5371 29212411 PMC5865621

[5] Harvey LA, Close JCT. Traumatic brain injury in older adults: characteristics, causes and consequences. Injury. 2012;43(11):1821–6. doi: 10.1016/j.injury.2012.07.188 22884759

[6] Albrecht JS, Liu X, Smith GS, Baumgarten M, Rattinger GB, Gambert SR, et al. Stroke incidence following traumatic brain injury in older adults. J Head Trauma Rehabil. 2015;30(2):E62-7. doi: 10.1097/HTR.0000000000000035 24816156 PMC4524572

[7] Bombardier CH, Hoekstra T, Dikmen S, Fann JR. Depression trajectories during the first year after traumatic brain injury. J Neurotrauma. 2016;33(23):2115–24. doi: 10.1089/neu.2015.4349 26979826 PMC5124751

[8] Deb S, Lyons I, Koutzoukis C, Ali I, McCarthy G. Rate of psychiatric illness 1 year after traumatic brain injury. Am J Psychiatry. 1999;156(3):374–8. doi: 10.1176/ajp.156.3.374 10080551

[9] Gardner RC, Byers AL, Barnes DE, Li Y, Boscardin J, Yaffe K. Mild TBI and risk of Parkinson disease: a chronic effects of neurotrauma consortium study. Neurology. 2018;90(20):e1771–9. doi: 10.1212/WNL.0000000000005522 29669907 PMC5957305

[10] Barnes DE, Kaup A, Kirby KA, Byers AL, Diaz-Arrastia R, Yaffe K. Traumatic brain injury and risk of dementia in older veterans. Neurology. 2014;83(4):312–9. doi: 10.1212/WNL.0000000000000616 24966406 PMC4115602

[11] Albrecht JS, Wickwire EM. Sleep disturbances among older adults following traumatic brain injury. Int Rev Psychiatry. 2020;32(1):31–8. doi: 10.1080/09540261.2019.1656176 31547739 PMC6986451

[12] Bailey MD, Gambert S, Gruber-Baldini A, Guralnik J, Kozar R, Qato DM, et al. Traumatic brain injury and risk of long-term nursing home entry among older adults: an analysis of medicare administrative claims data. J Neurotrauma. 2023;40(1–2):86–93. doi: 10.1089/neu.2022.0003 35793112 PMC10162579

[13] Schneider ALC, Wang D, Gottesman RF, Selvin E. Prevalence of disability associated with head injury with loss of consciousness in adults in the United States: a population-based study. Neurology. 2021;97(2):e124–35. doi: 10.1212/WNL.0000000000012148 34039721 PMC8279570

[14] Selassie AW, Zaloshnja E, Langlois JA, Miller T, Jones P, Steiner C. Incidence of long-term disability following traumatic brain injury hospitalization, United States, 2003. J Head Trauma Rehabil. 2008;23(2):123–31. doi: 10.1097/01.HTR.0000314531.30401.39 18362766

[15] Harrison-Felix CL, Whiteneck GG, Jha A, DeVivo MJ, Hammond FM, Hart DM. Mortality over four decades after traumatic brain injury rehabilitation: a retrospective cohort study. Arch Phys Med Rehabil. 2009;90(9):1506–13. doi: 10.1016/j.apmr.2009.03.015 19735778

[16] Maas AIR, Menon DK, Manley GT, Abrams M, Åkerlund C, Andelic N, et al. Traumatic brain injury: progress and challenges in prevention, clinical care, and research. Lancet Neurol. 2022;21(11):1004–60. doi: 10.1016/S1474-4422(22)00309-X 36183712 PMC10427240

[17] Manley GT, Dams-O’Connor K, Alosco ML, Awwad HO, Bazarian JJ, Bragge P, et al. A new characterisation of acute traumatic brain injury: the NIH-NINDS TBI Classification and Nomenclature Initiative. Lancet Neurol. 2025;24(6):512–23. doi: 10.1016/S1474-4422(25)00154-1 40409315

[18] Bergen G, Stevens MR, Burns ER. Falls and fall injuries among adults aged >/=65 years - United States, 2014. MMWR Morbidity and Mortality Weekly Report. 2016;65(37):993–8. doi: 10.15585/mmwr.mm6537a2 27656914

[19] Mosenthal AC, Lavery RF, Addis M, Kaul S, Ross S, Marburger R, et al. Isolated traumatic brain injury: age is an independent predictor of mortality and early outcome. J Trauma. 2002;52(5):907–11. doi: 10.1097/00005373-200205000-00015 11988658

[20] Mosenthal AC, Livingston DH, Lavery RF, Knudson MM, Lee S, Morabito D, et al. The effect of age on functional outcome in mild traumatic brain injury: 6-month report of a prospective multicenter trial. J Trauma. 2004;56(5):1042–8. doi: 10.1097/01.ta.0000127767.83267.33 15179244

[21] Utomo WK, Gabbe BJ, Simpson PM, Cameron PA. Predictors of in-hospital mortality and 6-month functional outcomes in older adults after moderate to severe traumatic brain injury. Injury. 2009;40(9):973–7. doi: 10.1016/j.injury.2009.05.034 19540490

[22] Dams-O’Connor K, Gibbons LE, Landau A, Larson EB, Crane PK. Health problems precede traumatic brain injury in older adults. J Am Geriatr Soc. 2016;64(4):844–8. doi: 10.1111/jgs.14014 26925541 PMC5021441

[23] Albrecht JS, Gardner RC, Wiebe D, Bahorik A, Xia F, Yaffe K. Comparison groups matter in traumatic brain injury research: an example with dementia. J Neurotrauma. 2022;39(21–22):1518–23. doi: 10.1089/neu.2022.0107 35611968

[24] Kumar RG, Juengst SB, Wang Z, Dams-OʼConnor K, Dikmen SS, OʼNeil-Pirozzi TM, et al. Epidemiology of comorbid conditions among adults 50 years and older with traumatic brain injury. J Head Trauma Rehabil. 2018;33(1):15–24. doi: 10.1097/HTR.0000000000000273 28060201

[25] Kumar RG, Olsen J, Juengst SB, Dams-OʼConnor K, OʼNeil-Pirozzi TM, Hammond FM, et al. Comorbid conditions among adults 50 years and older with traumatic brain injury: examining associations with demographics, healthcare utilization, institutionalization, and 1-year outcomes. J Head Trauma Rehabil. 2019;34(4):224–32. doi: 10.1097/HTR.0000000000000470 30829819 PMC10234605

[26] Malec JF, Ketchum JM, Hammond FM, Corrigan JD, Dams-OʼConnor K, Hart T, et al. Longitudinal effects of medical comorbidities on functional outcome and life satisfaction after traumatic brain injury: an individual growth curve analysis of NIDILRR traumatic brain injury model system data. J Head Trauma Rehabil. 2019;34(5):E24–35. doi: 10.1097/HTR.0000000000000459 30829813 PMC6711835

[27] Albrecht JS, Chen C, Falvey JR. Trajectories of recovery following traumatic brain injury among older medicare beneficiaries. J Neurotrauma. 2024;41(21–22):2377–84. doi: 10.1089/neu.2023.0502 38279868 PMC11631801

[28] Papa L, Mendes ME, Braga CF. Mild traumatic brain injury among the geriatric population. Curr Transl Geriatr Exp Gerontol Rep. 2012;1(3):135–42. doi: 10.1007/s13670-012-0019-0 23589783 PMC3625036

[29] Silverberg ND, Iverson GL, Cogan A, Dams OCK, Delmonico R, Graf MJP, et al. The American Congress of Rehabilitation Medicine diagnostic criteria for mild traumatic brain injury. Arch Phys Med Rehabil. 2023;104(8):1343–55. doi: 10.1016/j.apmr.2023.03.036 37211140

[30] Randolph C. RBANS Update: Repeatable Battery for the Assessment of Neuropsychological Status. Bloomington, MN: Pearson; 2012.

[31] Randolph C, Tierney MC, Mohr E, Chase TN. The repeatable battery for the assessment of neuropsychological status (RBANS): preliminary clinical validity. J Clin Exp Neuropsychol. 1998;20(3):310–9. doi: 10.1076/jcen.20.3.310.823 9845158

[32] Guralnik JM, Simonsick EM, Ferrucci L, Glynn RJ, Berkman LF, Blazer DG, et al. A short physical performance battery assessing lower extremity function: association with self-reported disability and prediction of mortality and nursing home admission. J Gerontol. 1994;49(2):M85-94. doi: 10.1093/geronj/49.2.m85 8126356

[33] Dite W, Temple VA. A clinical test of stepping and change of direction to identify multiple falling older adults. Arch Phys Med Rehabil. 2002;83(11):1566–71. doi: 10.1053/apmr.2002.35469 12422327

[34] Bastien CH, Vallières A, Morin CM. Validation of the Insomnia Severity Index as an outcome measure for insomnia research. Sleep Med. 2001;2(4):297–307. doi: 10.1016/s1389-9457(00)00065-4 11438246

[35] Buysse DJ, Reynolds CF 3rd, Monk TH, Berman SR, Kupfer DJ. The Pittsburgh Sleep Quality Index: a new instrument for psychiatric practice and research. Psychiatry Res. 1989;28(2):193–213. doi: 10.1016/0165-1781(89)90047-4 2748771

[36] Yue JK, Vassar MJ, Lingsma HF, Cooper SR, Okonkwo DO, Valadka AB, et al. Transforming research and clinical knowledge in traumatic brain injury pilot: multicenter implementation of the common data elements for traumatic brain injury. J Neurotrauma. 2013;30(22):1831–44. doi: 10.1089/neu.2013.2970 23815563 PMC3814815

[37] Corrigan JD, Bogner J. Initial reliability and validity of the Ohio State University TBI Identification Method. J Head Trauma Rehabil. 2007;22(6):318–29. doi: 10.1097/01.HTR.0000300227.67748.77 18025964

[38] Hicks R, Giacino J, Harrison-Felix C, Manley G, Valadka A, Wilde EA. Progress in developing common data elements for traumatic brain injury research: version two--the end of the beginning. J Neurotrauma. 2013;30(22):1852–61. doi: 10.1089/neu.2013.2938 23725058 PMC3814822

[39] Bush K, Kivlahan DR, McDonell MB, Fihn SD, Bradley KA. The AUDIT alcohol consumption questions (AUDIT-C): an effective brief screening test for problem drinking. Arch Intern Med. 1998;158(16):1789–95. doi: 10.1001/archinte.158.16.1789 9738608

[40] Jorm AF. A short form of the Informant Questionnaire on Cognitive Decline in the Elderly (IQCODE): development and cross-validation. Psychol Med. 1994;24(1):145–53. doi: 10.1017/s003329170002691x 8208879

[41] Dubois B, Slachevsky A, Litvan I, Pillon B. The FAB: a frontal assessment battery at bedside. Neurology. 2000;55(11):1621–6. doi: 10.1212/wnl.55.11.1621 11113214

[42] Katz S, Downs TD, Cash HR, Grotz RC. Progress in development of the index of ADL. Gerontologist. 1970;10(1):20–30. 5420677 10.1093/geront/10.1_part_1.20

[43] Lawton MP, Brody EM. Assessment of older people: self-maintaining and instrumental activities of daily living. Gerontologist. 1969;9(3):179–86. doi: 10.1093/geront/9.3_part_1.179 5349366

[44] Wilson L, Boase K, Nelson LD, Temkin NR, Giacino JT, Markowitz AJ, et al. A Manual for the Glasgow Outcome Scale-extended interview. J Neurotrauma. 2021;38(17):2435–46. doi: 10.1089/neu.2020.7527 33740873 PMC8390784

[45] Sheikh JI, Y JA. Geriatric Depression Scale (GDS): Recent evidence and development of a shorter version. Clin Gerontol: The J Aging Mental Health. 1986;5(1–2):165–73.

[46] Zigmond AS, Snaith RP. The hospital anxiety and depression scale. Acta Psychiatr Scand. 1983;67(6):361–70. doi: 10.1111/j.1600-0447.1983.tb09716.x 6880820

[47] Bogner J, Corrigan JD. Reliability and predictive validity of the Ohio State University TBI identification method with prisoners. J Head Trauma Rehabil. 2009;24(4):279–91. doi: 10.1097/HTR.0b013e3181a66356 19625867

[48] Gontkovsky ST, Beatty WW, Mold JW. Repeatable battery for the assessment of neuropsychological status in a normal, geriatric sample. Clinical Gerontologist. 2004;27(3):79–86. doi: 10.1300/j018v27n03_07

[49] Lippa SM, Hawes S, Jokic E, Caroselli JS. Sensitivity of the RBANS to acute traumatic brain injury and length of post-traumatic amnesia. Brain Inj. 2013;27(6):689–95. doi: 10.3109/02699052.2013.771793 23672444

[50] Volpato S, Cavalieri M, Sioulis F, Guerra G, Maraldi C, Zuliani G, et al. Predictive value of the Short Physical Performance Battery following hospitalization in older patients. J Gerontol A Biol Sci Med Sci. 2011;66(1):89–96. doi: 10.1093/gerona/glq167 20861145 PMC3011958

[51] Guralnik JM, Ferrucci L, Pieper CF, Leveille SG, Markides KS, Ostir GV, et al. Lower extremity function and subsequent disability: consistency across studies, predictive models, and value of gait speed alone compared with the short physical performance battery. J Gerontol A Biol Sci Med Sci. 2000;55(4):M221-31. doi: 10.1093/gerona/55.4.m221 10811152 PMC12149745

[52] Tinetti ME, Doucette J, Claus E, Marottoli R. Risk factors for serious injury during falls by older persons in the community. J Am Geriatr Soc. 1995;43(11):1214–21. doi: 10.1111/j.1532-5415.1995.tb07396.x 7594154

[53] Tinetti ME, Speechley M, Ginter SF. Risk factors for falls among elderly persons living in the community. N Engl J Med. 1988;319(26):1701–7. doi: 10.1056/NEJM198812293192604 3205267

[54] Shumway-Cook A, Baldwin M, Polissar NL, Gruber W. Predicting the probability for falls in community-dwelling older adults. Phys Ther. 1997;77(8):812–9. doi: 10.1093/ptj/77.8.812 9256869

[55] Studenski S, Duncan PW, Chandler J, Samsa G, Prescott B, Hogue C, et al. Predicting falls: the role of mobility and nonphysical factors. J Am Geriatr Soc. 1994;42(3):297–302. doi: 10.1111/j.1532-5415.1994.tb01755.x 8120315

[56] Wickwire EM, Schnyer DM, Germain A, Williams SG, Lettieri CJ, McKeon AB, et al. Sleep, sleep disorders, and circadian health following mild traumatic brain injury in adults: review and research agenda. J Neurotrauma. 2018;35(22):2615–31. doi: 10.1089/neu.2017.5243 29877132 PMC6239093

[57] Carney CE, Buysse DJ, Ancoli-Israel S, Edinger JD, Krystal AD, Lichstein KL, et al. The consensus sleep diary: standardizing prospective sleep self-monitoring. Sleep. 2012;35(2):287–302. doi: 10.5665/sleep.1642 22294820 PMC3250369

[58] Fictenberg NL, Putnam SH, Mann NR, Zafonte RD, Millard AE. Insomnia screening in postacute traumatic brain injury: utility and validity of the Pittsburgh Sleep Quality Index. Am J Phys Med Rehabil. 2001;80(5):339–45. doi: 10.1097/00002060-200105000-00003 11327555

[59] Kaufmann CN, Orff HJ, Moore RC, Delano-Wood L, Depp CA, Schiehser DM. Psychometric characteristics of the insomnia severity index in veterans with history of traumatic brain injury. Behav Sleep Med. 2019;17(1):12–8. doi: 10.1080/15402002.2016.1266490 28098495 PMC5740012

[60] Theadom A, Cropley M, Parmar P, Barker-Collo S, Starkey N, Jones K, et al. Sleep difficulties one year following mild traumatic brain injury in a population-based study. Sleep Med. 2015;16(8):926–32. doi: 10.1016/j.sleep.2015.04.013 26138280

[61] Chen P-Y, Tsai P-S, Chen N-H, Chaung L-P, Lee C-C, Chen C-C, et al. Trajectories of sleep and its predictors in the first year following traumatic brain injury. J Head Trauma Rehabil. 2015;30(4):E50–5. doi: 10.1097/HTR.0000000000000086 25119653

[62] Walker JM, Mulatya C, Hebert D, Wilson SH, Lindblad AS, Weaver LK. Sleep assessment in a randomized trial of hyperbaric oxygen in U.S. service members with post concussive mild traumatic brain injury compared to normal controls. Sleep Med. 2018;51:66–79. doi: 10.1016/j.sleep.2018.06.006 30099354

[63] Wickwire EM, Williams SG, Roth T, Capaldi VF, Jaffe M, Moline M, et al. Sleep, sleep disorders, and mild traumatic brain injury. what we know and what we need to know: findings from a national working group. Neurotherapeutics. 2016;13(2):403–17. doi: 10.1007/s13311-016-0429-3 27002812 PMC4824019

[64] Bjelland I, Dahl AA, Haug TT, Neckelmann D. The validity of the Hospital Anxiety and Depression Scale. An updated literature review. J Psychosom Res. 2002;52(2):69–77. doi: 10.1016/s0022-3999(01)00296-3 11832252

[65] Whelan-Goodinson R, Ponsford J, Schönberger M. Validity of the Hospital Anxiety and Depression Scale to assess depression and anxiety following traumatic brain injury as compared with the Structured Clinical Interview for DSM-IV. J Affect Disord. 2009;114(1–3):94–102. doi: 10.1016/j.jad.2008.06.007 18656266

[66] Schönberger M, Ponsford J. The factor structure of the Hospital Anxiety and Depression Scale in individuals with traumatic brain injury. Psychiatry Res. 2010;179(3):342–9. doi: 10.1016/j.psychres.2009.07.003 20483471

[67] King NS, Crawford S, Wenden FJ, Moss NE, Wade DT. The Rivermead Post Concussion Symptoms Questionnaire: a measure of symptoms commonly experienced after head injury and its reliability. J Neurol. 1995;242(9):587–92. doi: 10.1007/BF00868811 8551320

[68] Maas AIR, Hukkelhoven CWPM, Marshall LF, Steyerberg EW. Prediction of outcome in traumatic brain injury with computed tomographic characteristics: a comparison between the computed tomographic classification and combinations of computed tomographic predictors. Neurosurgery. 2005;57(6):1173–82; discussion 1173-82. doi: 10.1227/01.neu.0000186013.63046.6b 16331165

[69] Curran PJ, Obeidat K, Losardo D. Twelve frequently asked questions about growth curve modeling. J Cogn Dev. 2010;11(2):121–36. doi: 10.1080/15248371003699969 21743795 PMC3131138

[70] Albrecht JS, Scherf A, Ryan KA, Falvey JR. Impact of dementia and socioeconomic disadvantage on days at home after traumatic brain injury among older Medicare beneficiaries: A cohort study. Alzheimers Dement. 2024;20(4):2364–72. doi: 10.1002/alz.13666 38294135 PMC11032564

